# Rare coding variant analysis in a large cohort of Ashkenazi Jewish families with inflammatory bowel disease

**DOI:** 10.1007/s00439-018-1927-7

**Published:** 2018-08-22

**Authors:** E. R. Schiff, M. Frampton, N. Ben-Yosef, B. E. Avila, F. Semplici, N. Pontikos, S. L. Bloom, S. A. McCartney, R. Vega, L. B. Lovat, E. Wood, A. Hart, E. Israeli, D. Crespi, M. A. Furman, S. Mann, C. D. Murray, A. W. Segal, A. P. Levine

**Affiliations:** 1 Centre for Molecular Medicine, Division of Medicine, University College London, London, UK; 2 Inflammatory Bowel Disease Unit, Institute of Gastroenterology and Liver Diseases, Hadassah-Hebrew University Medical Center, Jerusalem, Israel; 3 Medical and Population Genetics, Broad Institute, Cambridge, MA USA; 4 Analytical and Translational Genetics Unit, Massachusetts General Hospital, Boston, MA USA; 5 UCL Genetics Institute, Division of Biosciences, University College London, London, UK; 6 Department of Gastroenterology, University College London Hospital, London, UK; 7 Research Department of Tissue and Energy, Division of Surgery and Interventional Science, University College London, London, UK; 8 Gastroenterology Department, Homerton University Hospital, London, UK; 9 Gastroenterology Department, St Mark’s Hospital, London, UK; 10 Centre for Paediatric Gastroenterology, Royal Free Hospital, London, UK; 11 Gastroenterology Department, Barnet General Hospital, London, UK; 12 Centre for Gastroenterology, Royal Free Hospital, London, UK

## Abstract

Rare variants are thought to contribute to the genetics of inflammatory bowel disease (IBD), which is more common amongst the Ashkenazi Jewish (AJ) population. A family-based approach using exome sequencing of AJ individuals with IBD was employed with a view to identify novel rare genetic variants for this disease. Exome sequencing was performed on 960 Jewish individuals including 513 from 199 multiplex families with up to eight cases. Rare, damaging variants in loci prioritized by linkage analysis and those shared by multiple affected individuals within the same family were identified. Independent evidence of association of each variant with disease was assessed. A number of candidate variants were identified, including in genes involved in the immune system. The ability to achieve statistical significance in independent case/control replication data was limited by power and was only achieved for variants in the well-established Crohn’s disease gene, *NOD2*. This work demonstrates the challenges of identifying disease-associated rare damaging variants from exome data, even amongst a favorable cohort of familial cases from a genetic isolate. Further research of the prioritized rare candidate variants is required to confirm their association with the disease.

**Electronic supplementary material:**

The online version of this article (10.1007/s00439-018-1927-7) contains supplementary material, which is available to authorized users.

## Introduction

Crohn’s disease (CD) and ulcerative colitis (UC) are the two major forms of the inflammatory bowel diseases (IBD), a heterogeneous group of chronic and debilitating disorders involving inflammation of the gastrointestinal tract. The etiology of IBD involves an aberrant immune response to commensal microflora in genetically susceptible individuals (Malik 2015; Segal 2016). A positive family history remains the strongest risk factor for IBD, evidenced by epidemiological and genetic studies. The first gene associated with CD was *NOD2* (Hugot et al. 2001; Ogura et al. 2001). To date, genome-wide association studies (GWAS) have identified over 240 risk loci for IBD (Anderson et al. 2011; Barrett et al. 2008; Franke et al. 2010; Jostins et al. 2012; Liu et al. 2015; Mirkov et al. 2017; de Lange et al. 2017). While the identified risk loci and underlying genetic associations have informed our understanding of the etiopathogenesis of IBD, the variance explained by these risk loci does not account for the estimated heritability of the disease.

Missing heritability is posited to be found in rare, high-impact coding variants (Manolio et al. 2009). Such variants are difficult to identify in population-based exome sequencing studies due to limited power and the high baseline rate of rare, neutral variants (Zuk et al. 2014; Kosmicki et al. 2016). In the context of IBD, studying the Ashkenazi Jewish (AJ) population has the potential to facilitate rare variant identification because this population has a relatively high prevalence of IBD, at least fourfold that of non-Jewish Europeans (Calkins and Mendeloff 1986; Odes et al. 1989; Mayberry et al. 1986; Roth et al. 1989; Bernstein et al. 2006). Furthermore, the AJs are a genetically isolated population (Carmi et al. 2014), characterized by repeated bottlenecks, expansions and endogamy with a consequential reduction in genetic heterogeneity (Ostrer 2001). Additional advantage can be gained by studying cases which are familial (Zielinski et al. 2012) or of an extreme phenotype, e.g., early onset cases (Uhlig and Schwerd 2016) as they are thought to be enriched for functional causal variants with stronger effects.

The increased incidence of CD amongst AJs has been genetically interrogated through GWAS (Kenny et al. 2012), which highlighted five novel CD-associated loci, and more recently by the analysis of exome sequence data from 1855 AJ CD cases and 3044 AJ non-IBD controls (Rivas et al. 2018). AJ enriched CD risk alleles were observed in the well-established CD risk gene *NOD2* and in the gene *LRRK2*. Furthermore, AJ CD cases and controls were found to have a greater CD polygenic risk score (incorporating 124 CD risk alleles but not those in *NOD2* or *LRRK2*) compared with non-Jewish European individuals. The authors conclude the presence of a coordinated selection for both rare and common CD risk alleles in the AJ population (Rivas et al. 2018).

We recently demonstrated the utility of an AJ family-based approach for identifying possible causal rare variants for IBD (Levine et al. 2016) through the study of the two largest AJ multiplex families described to date (54 and 26 CD cases, respectively). A novel frameshift mutation in *CSF2RB* was identified which replicated in an independent AJ case/control cohort and was shown to cause a loss of function in vitro (Chuang et al. 2016).

With a view to further delineating the genetics of IBD, and in particular, to search for high-impact rare variants, we performed whole exome sequencing on a newly established cohort of 960 Jewish individuals from 199 small to medium sized multiplex families with IBD and sporadic cases. We employed linkage analysis and the prioritization of rare variants shared by multiple affected individuals within the same family. Independent evidence of replication of the association of each variant with IBD was assessed with correction for multiple testing. The well-established gene, *NOD2*, was prioritized along with a number of other candidates. Despite the theoretical advantages afforded by studying AJ families, the identification of rare variants for IBD proves challenging.

## Methods

### Ethics

As per previously (Levine et al. 2016), ethical research governance approval was provided by the National Research Ethics Service London Surrey Borders Committee (10/H0906/115) and the University College London Research Ethics Committee (6054/001). Written informed consent was obtained from all participants.

### Cohort summary

A large cohort of AJ individuals with IBD were recruited, primarily in the United Kingdom, through advertisements, hospitals and primary care (Schiff et al. 2018). Additional family members were recruited through the probands. Participants were interviewed by telephone to ascertain their Jewish ancestry, IBD phenotype, age of diagnosis and family history of IBD both in first-degree and more distant relatives. Written confirmation of each affected individual’s diagnosis was obtained from his or her doctor in the majority of cases. Saliva samples were collected by post and DNA was isolated according to standard procedures (Quinque et al. 2006).

**Fig. 1:**
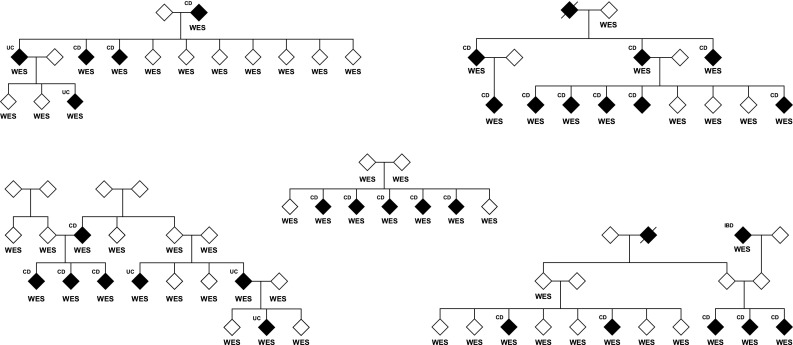

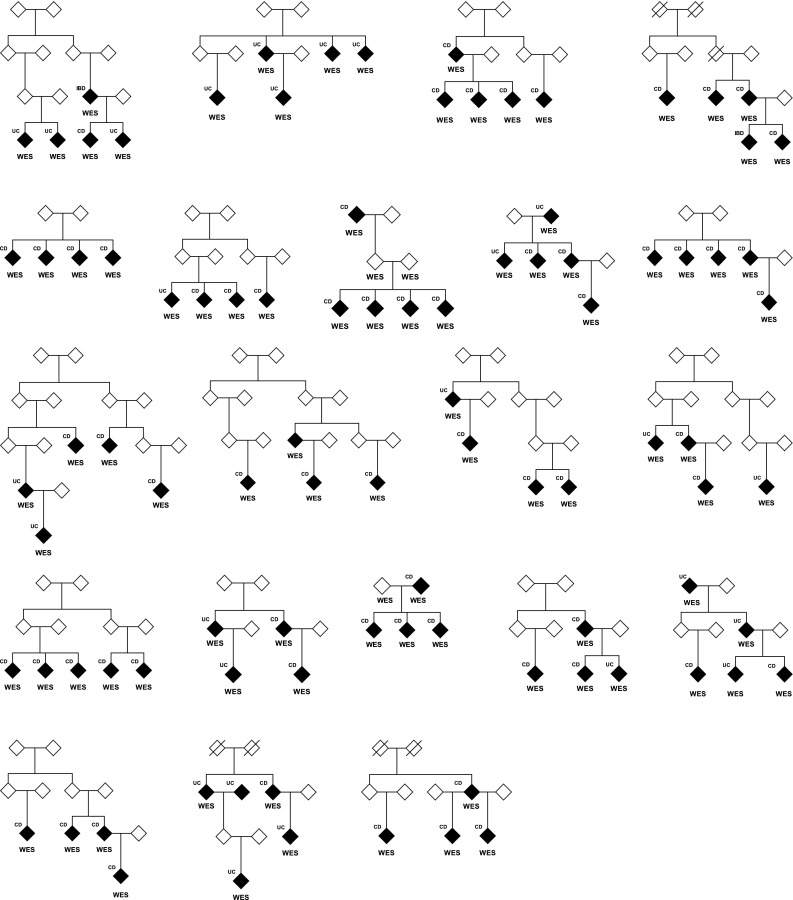
Pedigrees for the 26 largest families (lfams) with four or more exome sequenced affected individuals (WES). Affected individuals are indicated by filled symbols. Deceased individuals are indicated by a diagonal line. Phenotype (IBD, CD or UC) is shown. **a** Five families with two or more exome sequenced unaffected individuals. **b** 21 families with no exome sequenced unaffected individuals

Whole exome sequencing was performed on 960 individuals comprising 513 cases from 199 multiplex IBD families, 364 sporadic cases and 83 unaffected individuals (predominantly relatives of affected individuals). The familial cases consisted of 340 individuals with CD, 160 with UC and 13 with an unknown or unclassified IBD subtype (IBD-U). The sporadic cases consisted of 205 individuals with CD, 153 with UC and 6 with IBD-U. Pedigrees for the 26 largest families with at least four affected individuals (lfams) are shown in Fig. 1. The number of exome sequenced affected individuals with IBD or CD per family are shown in Supplementary Table 1.

### Whole-exome sequencing

#### Data generation

Indexed paired-end libraries were prepared using the BGI 59 Exome Enrichment Kit (BGI, China) or the Agilent SureSelect Exome v4 Kit (Agilent, USA), and 2 × 150 bp sequencing was performed by BGI or Macrogen (Macrogen, South Korea) on the Illumina HiSeq 2000 system (Illumina, USA). Two samples were sequenced by both platforms and variant calls were 99.8% concordant (Supplementary Fig. 1).

#### Read alignment and variant calling

Sequence read alignment and variant calling was performed alongside 4325 other exomes (UCLex) using an in-house next-generation sequencing analysis pipeline (Pontikos et al. 2017). Reads were aligned to the human reference genome build 37/hg19 by Novoalign (version 3.02.08) and variants were called using the Genome Analysis Toolkit (GATK) (McKenna et al. 2010) according to best practices with local realignment around indels, followed by joint variant calling and variant quality score recalibration (VQSR) (DePristo et al. 2011). VQSR uses machine learning to calculate quality scores for variants considering a number of quality parameters and employing a training set of highly validated variants as likely true positives. Further requirements for quality assurance included: genotyping quality (GQ) ≥ 30, reference/alternate read depth for heterozygote calls approximates to a 1:1 ratio (Chi-squared test *p* ≥ 0.001), alternate read depth for heterozygote and homozygote calls ≥ 3 and VQSR truth tranche ≤ 99.5% for SNPs and ≤ 99.0% for indels. In addition, variants were required to have a call rate ≥ 0.75 and to approximate to Hardy Weinberg Equilibrium (HWE) (chi-squared test, *p* ≥ 5 × 10^− 8^).

#### Variant annotation and filtering

The variant effect predictor (VEP version 76) (McLaren et al. 2016) was used to annotate all variants for their predicted impact on Ensembl gene transcripts and their frequency in Ashkenazi Jewish (gnomAD-AJ) individuals (*n* = 4925) and non-Finnish European (gnomAD-NFE) individuals (*n* = 55,860) from the gnomAD exome database (Lek et al. 2016) (http://gnomad.broadinstitute.org). Missense variants were annotated for their predicted deleteriousness according to CAROL (Lopes et al. 2012). Custom-made scripts were used to further annotate each variant with their CADD score (Kircher et al. 2014) and their frequency in a cohort of 500 non-IBD unrelated individuals from amongst the jointly processed UCLex samples that were most proximal to the sequenced AJs based on principal component analysis (UCLex-pAJ), as defined below. A variant was considered potentially pathogenic, and hence included, if it was a frameshift, start loss, stop gain, splice site acceptor/donor variant, or a missense variant which was predicted to be damaging by CAROL and had a CADD score ≥ 20. Variants were filtered based on their population frequency using all of gnomAD-AJ, gnomAD-NFE and UCLex-pAJ; variants were considered as either very rare (AF < 0.005), rare (AF < 0.05) or common (AF > 0.05). If the reference genome carried the minor allele, then genotypes and population allele frequencies were flipped.

### Quality control

#### Sex

The ascertained sex of each sample was verified by the homozygosity rate of common variants on the non-pseudoautosomal regions of the X chromosome (Supplementary Fig. 2).

#### Relatedness

KING (Manichaikul et al. 2010) was used to calculate a kinship coefficient for each pair of samples based on common variants to identify duplicates and verify the ascertained family pedigrees. Kinship coefficients were mapped to relationships using default thresholds as follows: > 0.354 for duplicate samples/monozygotic twins, 0.177–0.354 for first-degree relatives, 0.0884–0.177 for second-degree relatives, 0.0442–0.0884 for third-degree relatives, and < 0.0442 for unrelated. In addition, a set of unrelated cases and a set of unrelated non-IBD UCLex samples were identified for use in ancestry quality control. A subset of the latter (UCLex-pAJ) was used for variant prioritization and gene burden testing (see “Ancestry” section).

#### Ancestry

The ascertained ancestry of samples was examined using principal component analysis (PCA) (Supplementary Fig. 3) with the R package FactoMineR (Lê et al. 2008). PCs were calculated from 5409 common independent exome-wide SNPs (Purcell et al. 2014) in 4602 unrelated UCLex samples. These samples include 1092 individuals from the 1000 Genomes Project (1000 Genomes Project Consortium et al. 2015) and 582 unrelated individuals from our cohort (as defined above). The remaining individuals in our cohort were projected onto the calculated PCs.

A subset of the recruited subjects was genetically defined as being of AJ ancestry as follows. The 920 individuals of self-declared AJ ancestry (excluding 40 from the recruited subjects of Sephardi, Middle Eastern (Ostrer 2001) or mixed Jewish ancestry) were defined by the first five PCs, multivariate outliers were removed and a five-dimensional Gaussian model fitted. The number of PCs and the confidence region used were varied and the false positive rate tested. A 90% ellipsoid confidence region captured 878 AJ individuals with only 17 false positives: sixteen that self-declared as being of Sephardi or Middle Eastern Jewish ancestry and one non-Jewish individual. The Mahalanobis distance from each non-IBD UCLex sample to these now genetically defined AJs was calculated to identify the 500 most proximal ancestry-matched controls (UCLex-pAJ).

### Analysis approach

The prioritization of candidate variants for IBD in AJ multiplex families was performed by employing two principal strategies. The first sought to identify variants present in affected individuals across multiple families. For this, linkage analysis of all the families in the cohort was performed separately for the phenotypes of CD and IBD (encompassing CD, UC and IBD-U). UC was not examined separately owing to the smaller number of families with this phenotype. Loci with suggestive evidence of linkage were prioritized (LOD ≥ 1.5) and variants within these loci that were shared by the families contributing to the linkage signal were identified. As variants present across multiple families were, by definition, not expected to be exceedingly rare in the population, an allele frequency (AF) threshold of < 0.05 (5%) was employed. Independent evidence of association with IBD, CD or UC was assessed for in a replication cohort of unrelated AJ cases and controls. The second strategy sought to identify rarer variants across the whole exome that may only be found in a smaller subset of the families via a family-based rare variant prioritization analysis. For this, an AF threshold of < 0.005 (0.5%) was employed. As previously, independent evidence of association with IBD, CD or UC was assessed. Statistical evidence of an enrichment of the variant with disease amongst all the sequenced affected individuals in the families was further examined by gene dropping. Replication of such rare variants was limited by restrictive power (Kosmicki et al. 2016).

### Linkage analysis

The SNP map used for linkage was generated as follows. First, all common (AF > 0.05) SNPs were extracted, assigned a genetic map position from the Rutgers map (Matise et al. 2007) and pruned for linkage disequilibrium (LD) (window size 50 SNPs, step size 5, *r*^2^ threshold 0.2) in UCLex-pAJ using PLINK (Purcell et al. 2007). The remaining SNPs were assigned an AF using gnomAD-AJ. In each 0.3 centiMorgan (cM) window, only the SNP with maximum heterozygosity was retained using the lowest minor allele frequency (MAF) SNP to break ties. The resulting SNP map contained 5945 SNPs.

Only affected individuals within the families were considered. The pedigrees were trimmed to remove non-informative individuals using the R package kinship2 (Sinnwell et al. 2014) leaving 150 informative families for IBD and 88 for CD. A non-parametric linkage analysis was performed using MERLIN (Abecasis et al. 2002) employing NPL ALL which places additional weight on families with three or more affected individuals (Whittemore and Halpern 1994). The maximum theoretical LOD scores for IBD and CD were 65.6 and 38.4, respectively. Genes partially or fully within a region with a log odds (LOD) score ≥ 1.5 were selected. Although not at genome-wide significance (Lander and Kruglyak 1995), such loci demonstrate suggestive evidence of linkage and may harbor disease-related variants. The per-family LOD scores at these loci were computed. For each of the linkage prioritized genes, rare (AF < 0.05) damaging variants seen in at least two affected family members in at least two separate families that were contributing to the linkage (with mean LOD > 0 across the region of interest) were identified.

### Family-based rare variant prioritization and segregation analysis

With a view to identifying potentially pathogenic variants enriched in one or more family with the disease, 26 families with four or more CD or IBD (CD, UC and IBD-U) sequenced affected individuals (lfams) were considered (Fig. 1). Variants that were very rare in the population (AF < 0.005) and that were predicted to be damaging (as above) were included. A variant was retained if it was observed in at least 75% of the affected individuals in at least one family (CD or IBD separately).

The segregation of variants to unaffected individuals within five families with at least two sequenced unaffected carrier siblings or offspring for either CD or IBD was examined (Fig. 1a). Potential obligate carriers (parents of affected individuals) were excluded. The probabilities of the genotypes observed in these unaffected individuals, conditional on the observed genotypes in the affected individuals (assuming heterozygosity and only one carrier founder) were calculated. Variants observed in less than one-third of the unaffected individuals were prioritized.

### Case/control association for replication

Variants prioritized from both loci identified by linkage analysis and the family-based rare variant and segregation analyses were examined for evidence of association with IBD (*n* = 1867), CD (*n* = 1286) and UC (*n* = 544) as compared with controls (*n* = 3035–3616) in an independent cohort of genetically confirmed AJ individuals (Rivas et al. 2018). For each phenotype in this independent dataset (IBD, CD and UC), identity by descent filtering was employed to remove related samples, prioritizing the cases, resulting in a slightly different number of controls for each. Per variant quality control of these data was undertaken using gnomAD indicators including call rate and random forest based filtering. No data were available for variants for which the alternate allele was seen in only one case or control. Given the cohort sizes for each phenotype and alpha (*p* value threshold after correction for multiple testing, see below), power calculations were performed for a range of population AFs and effect sizes via a simulation approach which sampled log odds ratios and applied Fisher’s exact test.

### Gene dropping for replication

Gene dropping (MacCluer et al. 1986) was used to assess for an enrichment of variants prioritized from the family-based rare variant analysis in additional families in the study cohort relative to the control population. In each family, a genotype (number of copies of the alternate allele) was assigned to each founder using a Binomial distribution where the number of trials was two and the success probability in each trial was the control AF (maximum of gnomAD-AJ, gnomAD-NFE and UCLex-pAJ). If data were not available from gnomAD-AJ (exomes), the gnomAD AJ whole-genome dataset was utilized (*n* = 151). A depth-first traversal starting from each founder (one per spousal pair) was performed, and in the case of heterozygotes, a random number generator determined which parental allele was transmitted to each child. Thus, every individual in the family was assigned a genotype. Having performed this gene dropping across all the families, an overall simulated AF was calculated amongst all the cases excluding those from the family in which the variant was initially identified. The whole process was repeated 100,000 times, thus producing 100,000 simulated AFs. The probability of the variant being at a greater frequency in the family disease cohort relative to the simulated frequency under the null, and hence independent evidence of association with disease, was calculated.

Given the alpha value (*p* value threshold after correction for multiple testing, see below), power calculations were performed for CD and IBD for a range of population and cohort AFs as follows. First, cohort AFs were simulated using the population AF (*n* = 100,000), then the number of simulated frequencies less than the cohort frequency was modeled as a binomial distribution, and finally this binomial distribution was approximated to a normal distribution to extract power.

### Correction for multiple testing

A Bonferroni threshold correcting for multiple testing was calculated based on the number of variants prioritized from both the linkage analysis (*n* = 11) and the family-based rare variant analysis (*n* = 413). For the former, three datasets were examined (IBD, CD and UC case/control replication). For the latter, two additional CD and IBD gene dropping datasets were examined. Examining all 424 variants, there was a highly significant correlation (Spearman’s rank test) between the CD and IBD case/control replication *p* values (*p* = 3.7 × 10^− 8^) but not between CD and UC (*p* = 0.234) or UC and IBD (*p* = 0.094). Amongst the 413 variants for which gene dropping was performed, there was also a highly significant correlation between the CD and IBD *p* values (*p* < 2 × 10^− 16^). There was no significant correlation between the case/control replication and the gene dropping *p* values (*p* = 0.53 for IBD and *p* = 0.71 for CD). As a consequence of the observed significant correlations, the number of datasets examined for the purpose of calculating the Bonferroni threshold was defined as two from the case/control replication and one from the gene dropping. Thus, the total number of tests performed was considered 11 × 2 + 413 × 3 yielding a Bonferroni threshold of *p* < 3.97 × 10^− 5^.

### Gene burden testing

Gene burden analysis was performed to test for a statistically significant excess burden of rare damaging variants on a gene by gene basis amongst cases relative to controls. The cases utilized were the unrelated genetically defined AJ individuals (330 for CD and 550 for IBD). The controls were drawn from the UCLex-pAJ subjects with a case–control ratio of one (hence for IBD, the next 50 most proximal controls were added to UCLex-pAJ). For each gene, a combined multivariate and collapsing (CMC) test (Li and Leal 2008) was applied. Variants with AF < 0.01 in gnomAD-AJ were aggregated into one binary variable and variants with an AF > 0.01 and < 0.05 into another. If the variant was absent in the gnomAD-AJ population, then gnomAD-NFE or UCLex-pAJ frequencies were used. Only damaging variants were considered. The multivariate test was a log-likelihood ratio (LLR) test in which the null and alternative hypothesis models included covariates for the first five ancestry PCs. Given the observed inflation (Supplementary Fig. 4), these results were only used to provide supplementary data for prioritized variants.

## Results

### Linkage analysis

Non-parametric linkage analysis of IBD identified two loci achieving LOD ≥ 1.5 on chromosomes 9 and 13 (Supplementary Fig. 5A) with maximum LOD scores of 1.741 and 1.873, respectively. Linkage analysis of CD identified only one locus achieving LOD ≥ 1.5, on chromosome 16 with a maximum LOD score of 2.074 (Supplementary Fig. 5B). Supplementary Table 2 summarizes these linkage regions in terms of their genomic position and the genes they contain. Of note, *NOD2* is within the chromosome 16 locus.

The distribution of previously described CD-associated *NOD2* variants amongst the cohort is shown in Supplementary Table 3. The variant G908R (rs2066845) failed quality control owing to a poor call rate. All of the other well-established *NOD2* variants (Huang et al. 2017; Rivas et al. 2018) were identified in at least one family and their AF in the unrelated CD cases approximated to that in both the independent AJ case/control dataset and the literature (e.g., L1007insC/rs2066847 was observed at an AF of 0.075 in CD cases compared with 0.072 in CD cases in the AJ case/control dataset).

A total of 51 rare (AF < 0.05) damaging variants were observed in at least two individuals in at least one family in the genes within the linkage-defined loci. Of these, 30 variants were seen in a family contributing to the linkage (per-family LOD > 0) and 11 were seen in at least two such families (Table 1 and Supplementary Table 4) representing all three of the linkage loci. Replication data in unrelated AJ cases and controls were available for all of these variants. Examining IBD, CD and UC and employing the Bonferroni threshold of *p* < 3.97 × 10^− 5^ (see “Methods”), three variants, all within the gene *NOD2*, were significantly associated with disease. One additional *NOD2* variant approached the significance threshold (*p* = 3.7 × 10^− 4^). Of the eight remaining variants, none achieved *p* < 0.05 with a positive odds ratio. Their population control AFs ranged from 0.04 down to 0.004. At this AF range, OR of 1.7–3.6, 2.1–3.8 and 2.1–5.5 would be required for 80% power to achieve significance at the Bonferroni threshold in IBD, CD or UC, respectively.

**Table 1:** Rare (< 0.05) variants prioritized from linkage loci observed in at least two affected individuals in at least two families contributing to the linkage

Variant	rsID	Gene	Consequence	Effect	Control AF	Number of families	Replication			
							P	OR	Case AF	Control AF
16_50763778_G_GC	rs2066847	*NOD2*	Frameshift	L1007insC	0.035	3	2.7×10^-26^	3.24	0.078	0.024
16_50745656_G_A	rs104895438	*NOD2*	Missense	A612T	7.4×10^-3^	2	4.3×10^-10^	4.82	0.016	3.3×10^-3^
16_50745926_C_T	rs2066844	*NOD2*	Missense	R702W	0.043	6	3.4×10^-5^	1.79	0.037	0.021
16_50750810_A_G	rs104895467	*NOD2*	Missense	N852S	0.016	6	3.7×10^-4^	1.82	0.025	0.014
13_95863008_C_A	rs11568658	*ABCC4*	Missense	G187W	0.040	4	0.16	0.841	0.037	0.044
9_97869536_C_T	rs1800367	*FANCC*	Missense	V449M	4.1×10^-3^	2	0.18	1.69	5.1×10^-3^	3.1×10^-3^
9_97367834_G_A	rs200679026	*FBP1*	Missense	R244W	0.010	2	0.30	0.778	8.2×10^-3^	0.011
16_48130781_C_T	rs36102575	*ABCC12*	Stop	W1024Ter	0.033	2	0.37	1.19	0.015	0.013
16_48204130_C_T	rs60681475	*ABCC11*	Splice acceptor		0.020	4	0.56	1.11	0.021	0.019
16_49430534_G_A	rs72776789	*C16orf78*	Missense	E199K	0.042	3	1.0	1.01	0.044	0.043
16_50338341_C_T	rs61731915	*ADCY7*	Missense	A480V	0.020	3	1.0	1.01	0.020	0.020

Full results are shown in Supplementary Table 4

*Variant* chromosome and genome position (Build 37) of variant with the reference and alternative alleles, *Effect* HGVS protein alteration, *Control AF* maximum allele frequency of gnomAD-AJ, gnomAD-NFE and UCLex-pAJ, *Number of families* the number of families with at least two affected individuals with the variant that are contributing to the linkage (LOD > 0), *Replication* independent AJ case/control association results, data (*P p* value, *OR* odds ratio, *AF* allele frequency) are shown from the phenotype (IBD, CD or UC) achieving the minimum *p* value.

Examining the gene burden results for the eight prioritized unique genes in CD and IBD, only *NOD2* in CD was significant at *p* < 0.05 with *p* = 7.7 × 10^− 6^.

### Family-based rare variant prioritization

Examining very rare (AF < 0.005) damaging variants that were observed in at least 75% of the affected individuals in 26 families with four or more affected individuals (lfams) identified 413 variants (Supplementary Table 5). One variant was observed in two lfams, a missense in *NLRP9* (rs143301793). Of the 413 variants, 362 were seen in an lfam with IBD and 233 were seen in an lfam with CD (182 in common). The IBD variants were identified in 24 families with a median of 11.5 per family and a range of 1–51. The CD variants were identified in 13 families with a median of 12 per family and a range of 5–51.

Replication data in unrelated AJ cases and controls were available for 207 of the 413 variants; the remainder were absent owing to failure to pass quality control filtering or due to an alternate allele count of zero or one in the cases and controls examined. As expected, these 207 variants were more common than those for which replication data were missing (mean UCLex-pAJ AF 9.4 × 10^− 4^ versus 2.9 × 10^− 4^, Wilcoxon *p* = 9 × 10^− 11^). Examining IBD, CD and UC and employing the Bonferroni threshold of *p* < 3.97 × 10^− 5^, no variants were associated with disease. The variant with the smallest *p* value was a missense variant in the gene *DNAH3* (rs140821281, *p* = 0.0016). The population AFs for these variants ranged from zero to 4.99 × 10^− 3^. At an AF range of 4.99 × 10^− 3^ down to 9 × 10^− 6^ (the smallest non-zero value observed), OR of 3.3–20.5, 3.4–24.6 and 4.8–33.5 would be required for 80% power to achieve significance at the Bonferroni threshold in IBD, CD or UC, respectively.

In the gene dropping data, which examines for an excess of the variant in the familial cohort with the index family removed, two variants achieved the Bonferroni threshold. These were a missense variant in the gene *ZNF366* (S332R) and a splice donor variant in the gene *MDGA1* (rs202070332). They were each seen in affected individuals in one additional family within the cohort yielding maximum disease cohort AFs of approximately 0.0015, enriched from the population frequency (zero) at *p* < 1 × 10^− 5^ (100,000 simulations). No case/control replication data were available for either of these variants owing to their rarity.

At an AF range of 4.99 × 10^− 3^ down to 9 × 10^− 6^, the extent to which the AF in the family cohort would have to exceed the population control AF for 80% power to achieve significance at the Bonferroni threshold would be 0.005–0.0167 for IBD. Examining only CD cases in the families, the equivalent increase would have to be 0.006–0.0203.

The 233 and 362 variants seen in a CD or IBD lfam, respectively, were in 226 and 353 unique genes. Gene burden results were available for 187 and 305 of these, respectively. No gene achieved *p* < 1.4 × 10^− 4^ (correcting for 353 genes).

### Segregation analysis

Amongst five families each with two or more unaffected offspring or siblings of affected individuals, 68 variants were present in ≥ 75% of the cases (CD or IBD). Of these variants, nine were present in ≤ 1/3 of the relevant unaffected family members (Table 2) from four families. Pedigrees of these families showing the segregation of the prioritized variants are in Supplementary Fig. 6.

**Table 2:** Very rare (< 0.005) variants prioritized in four large families with at least two sequenced unaffected offspring or siblings of affected individuals, where ≤ 1/3 of the unaffected individuals carry a variant seen in at least 75% of the affected individuals

Variant	rsID	Gene	Consequence	Effect	Control AF	Affected	Unaffected
1_85462527_TG_T	rs748190234	*MCOLN2*	Frameshift	Q10X	2.42×10^-3^	5/5	1/5
2_197873690_C_A		*ANKRD44*	Missense	D656Y	0	6/8	1/3
6_128134406_C_G	rs141905910	*THEMIS*	Missense	K460N	1.93×10^-3^	6/8	1/3
8_1581122_C_G		*DLGAP2*	Missense	R494G	0	6/8	1/3
19_55496518_A_C	rs775798143	*NLRP2*	Missense	S712R	7.11×10^-4^	6/8	1/3
9_139286443_G_A	rs61731233	*SNAPC4*	Missense	A309V	3.28×10^-3^	5/5	3/9
19_41056172_C_T	rs145109616	*SPTBN4*	Missense	A1538V	3.13×10^-3^	4/5	3/9
11_61120544_G_T	rs201384498	*CYB561A3*	Missense	P171H	2.71×10^-3^	4/5	0/2
11_66009061_G_C	rs202231158	*PACS1*	Missense	G865R	4.98×10^-3^	4/5	0/2

Full results are shown in Supplementary Table 5

*Variant* chromosome and genome position (Build 37) of variant with the reference and alternative alleles, *Effect* HGVS protein alteration, *Control AF* maximum allele frequency of gnomAD-AJ, gnomAD-NFE and UCLex-pAJ, *Affected* proportion of the affected individuals in the family heterozygous for the variant, *Unaffected* proportion of the unaffected individuals in the family heterozygous for the variant.

None of these four families had power to detect a significant lack of transmission of variants to ≤ 1/3 of the unaffected family members conditional on the observed genotypes amongst the affected individuals at *p* < 0.05.

Replication data were available for six of these variants, none of which were significant at *p* < 0.05. The minimum gene dropping *p* value achieved was *p* = 0.016 for a missense variant in *CYB561A3*. The power to detect an association is as given above.

## Discussion

In this study, we have searched for rare coding variants for IBD using whole exome sequencing. This is a challenging endeavor because of the complex and polygenic nature of genetic susceptibility to IBD, itself a heterogeneous phenotype. To help address this complexity, we studied familial cases from the AJ population which, due to its population history, may harbor a smaller number of pathogenic variants compared with an outbred population. This is the first time a familial AJ IBD cohort of this size has been subjected to exome sequencing.

To identify rare, damaging variants potentially implicated in the aetiopathogenesis of the disease, we employed linkage analysis and selected variants observed in multiple members of the same family. The resulting prioritized variants were assessed for statistical evidence of association with IBD in an independent AJ case/control cohort and using gene dropping simulations. Through this approach, variants in the genes *NOD2, ZNF366* and *MDGA1* achieved statistical significance after correcting for multiple testing. *ZNF366* is of particular interest as it is a dendritic cell-specific transcription factor implicated in the regulation of IL10 (Søndergaard et al. 2018). A large number of additional very rare variants were identified as being enriched in familial cases but the replication cohort and gene dropping simulations lacked the power to confirm or refute an association given their low frequency. It is also relevant to note that the gene dropping significance relies heavily upon the observed control AF, the accuracy of which is limited by the sample size of the studied cohorts (Qiao et al. 2017) and furthermore that the observation of a second family sharing a very rare variant (such that the gene dropping is significant) could be confounded by cryptic relatedness.

Variants were further prioritized based on their lack of transmission to unaffected siblings and offspring; although of limited power to achieve statistical significance, this provided suggestive evidence. A number of these variants were of interest as the genes were either previously described in relation to IBD [such as *THEMIS* (Chabod et al. 2012)] or were involved in the regulation of the innate immune system which is of increasingly recognized importance in the pathogenesis of IBD (Sewell et al. 2012; Smith et al. 2009). These genes include *MCOLN2* (Cuajungco et al. 2016) and *NLRP2* (Bruey et al. 2004). Interestingly, we previously identified a different variant in *NLRP2* in another large AJ CD family (Levine et al. 2016).

Even with the advantages afforded by our study design, focusing on an isolated population and multiplex families, the robust identification of rare, damaging candidate variants proved challenging. This is likely secondary to technical/analytic deficiencies, and importantly, insufficient power. Given the AF thresholds imposed in our study, the number of variants prioritized and the size of the replication cohort, 80% power to achieve significance would only be observed for a variant with an OR exceeding 2 or 3.5 at the higher limit of population AFs for rare (AF < 0.05) and very rare (AF < 0.005) variants, respectively, with these numbers increasing substantially as the AF decreases towards zero. Considerably larger cohort sizes would be required to test for association at smaller effect sizes for such rare variants. Furthermore, when examining the lack of transmission of candidate variants to unaffected individuals within families, there was an insufficient number of meiosis and larger numbers of unaffected individuals within these families would be required.

The unequivocal identification of *NOD2* not only confirms its undoubtedly strong association with CD in this population but more importantly, it validates the methods used. The results also suggest that further damaging exonic single nucleotide risk variants for IBD in AJ multiplex families at AF > 0.01 and < 0.05 that are of comparable effect size to that of the *NOD2* variants are unlikely to be present.

This study has not examined the role of known IBD-associated common variants (AF > 0.05) (Mirkov et al. 2017) which are recognized to contribute substantially to the heritability of the disease, including in families (Stittrich et al. 2016). To fully evaluate the genetic architecture of IBD in this familial AJ cohort, such variants will need examination.

This study has demonstrated the challenges of identifying disease-associated rare damaging variants from exome data, even amongst a favorable cohort of familial cases from a genetic isolate. Further research of the prioritized rare candidate variants is required to test their association with the disease. A combination of environmental factors, common variants, and rare variants (some of which we may have identified) are likely to contribute to the familial aggregation of IBD in AJ families.

## Electronic supplementary material

Below is the link to the electronic supplementary material.

Supplementary Table Legends and Figures (PDF 931 KB)

Supplementary Table 1 (XLSX 15 KB)

Supplementary Table 2 (XLSX 12 KB)

Supplementary Table 3 (XLSX 15 KB)

Supplementary Table 4 (XLSX 16 KB)

Supplementary Table 5 (XLSX 127 KB)
