# Supplementary material for: Rare coding variant analysis in a large cohort of Ashkenazi Jewish families with inflammatory bowel disease
Source: Hum Genet. 2018 Aug 22;137(9):723–34. doi: 10.1007/s00439-018-1927-7 (PMC6153494; doi:10.1007/s00439-018-1927-7)

Rare coding variant analysis in a large cohort of Ashkenazi Jewish families with inflammatory bowel disease

E. R. Schiff and M. Frampton, N. Ben Yosef, B. E. Avila, F. Semplici, N. Pontikos, S. L. Bloom, S. A. McCartney, R. Vega, L. B. Lovat, E. Wood, A. Hart, E. Israeli, D. Crespi, M. A. Furman, S. Mann, C. D. Murray, A.W. Segal and A. P. Levine

Human Genetics

2018

https://doi.org/10.1007/s00439-018-1927-7

Supplementary Table Legends and Figures

	Page
Supplementary Table Legends	2
Supplementary Figure 1	3
Supplementary Figure 2	4
Supplementary Figure 3A	5
Supplementary Figure 3B	6
Supplementary Figure 4	7
Supplementary Figure 5A	8
Supplementary Figure 5B	9
Supplementary Figure 6	10

https://doi.org/10.1007/s00439-018-1927-7

Supplementary Table Legends

Supplementary Table 1
The number of exome sequenced affected individuals with IBD (IBD_n) or CD (CD_n) in the 199 families studied.

Supplementary Table 2
Three loci identified by non-parametric linkage analysis (LOD ≥ 1.5) of inflammatory bowel disease (IBD) on chromosomes 9 and 13 and Crohn's disease (CD) on chromosome 16. The genomic positions (Build 37) and the number and identity of the genes within each loci are shown.

Supplementary Table 3
Details for seven *NOD2* variants described in Rivas et al. [PMID 29795570] and Huang et al. [PMID 28658209] identified in our dataset including their frequency and the number of families with at least one affected individual with each variant. Separate sheets in the Excel document contain (i) the column definitions and (ii) the results.

Supplementary Table 4
Rare (AF < 0.05) variants prioritised from linkage loci observed in at least two affected individuals in at least two families contributing to the linkage, showing the full annotated results. Separate sheets in the Excel document contain (i) the column definitions and (ii) the results.

Supplementary Table 5
Very rare (AF < 0.005) variants prioritised as being in at least 75% of affected individuals with IBD or CD in at least one of the 26 large families (lfams). Separate sheets in the Excel document contain (i) the column definitions and (ii) the results.

https://doi.org/10.1007/s00439-018-1927-7

Supplementary Figure 1

Number and concordance of variant calls for the same sample sequenced across different sequencing platforms (Macrogen and BGI). (A) Number of variants calls. (B) Concordance rate of variant calls (percentage).

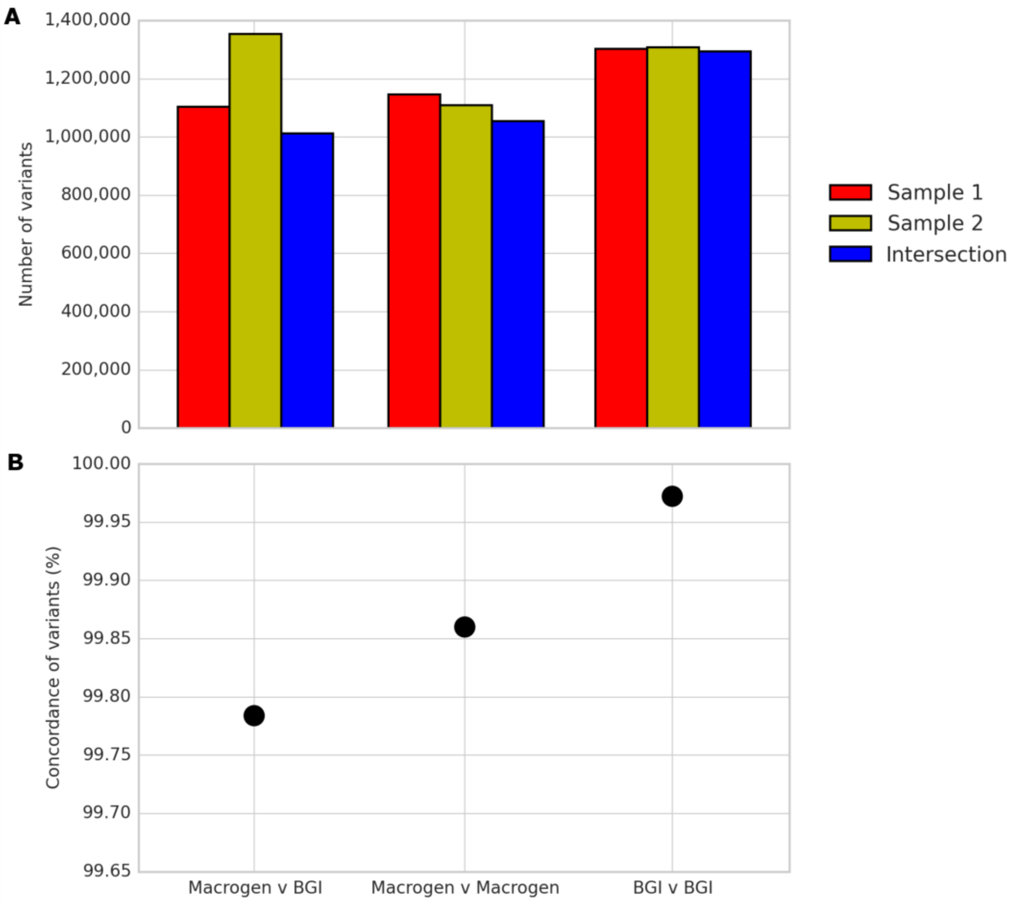

https://doi.org/10.1007/s00439-018-1927-7

Supplementary Figure 2

Homozygosity rate of common SNPs (minor allele frequency ≥ 0.05) on the non-pseudoautosomal regions of the X chromosome for each sample in our cohort. Samples are coloured according to their ascertained sex – blue for male and pink for female. For all samples, ascertained sex matches the sex inferred from the homozygosity rate.

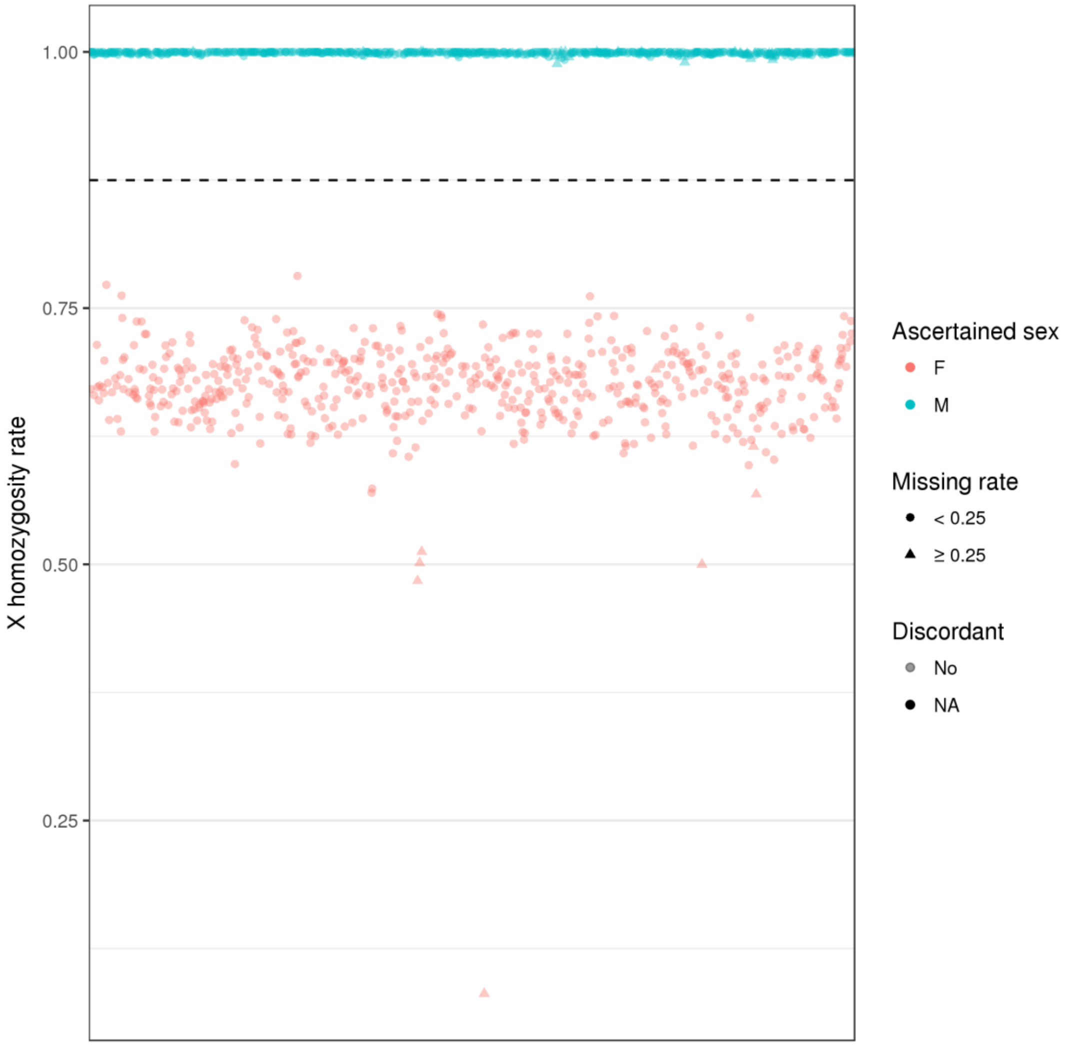

Supplementary Figure 3

Ancestry Principal Component Analysis (PCA) where PCs are calculated from common independent exome-wide SNPs and unrelated samples. (A) This plot represents 1000 Genomes Project (1KG) samples, and all samples in our cohort. Samples are coloured according to their ascertained ethnicity, which for the 1KG samples is: Northern and Western European (CEU), Southern Han Chinese (CHS) or Yoruba in Ibadan, Nigeria (YRI), and in our cohort: Ashkenazi Jewish (AJ), Non-Jewish (NJ), Partial-Jewish (PJ) or Sephardic or Middle Eastern Jewish (SJ). The unrelated UCLex samples used to calculate the PCs include 1,092 1000 Genomes Projects samples (from various European, East Asian and African populations) and 582 samples from our own cohort. The remaining individuals in our cohort were projected onto the PCs. (B) This plot represents the unrelated non-IBD UCLex samples on the same axes with the 500 individuals most proximal to the genetically defined AJ individuals (UCLex-pAJ) indicated.

A

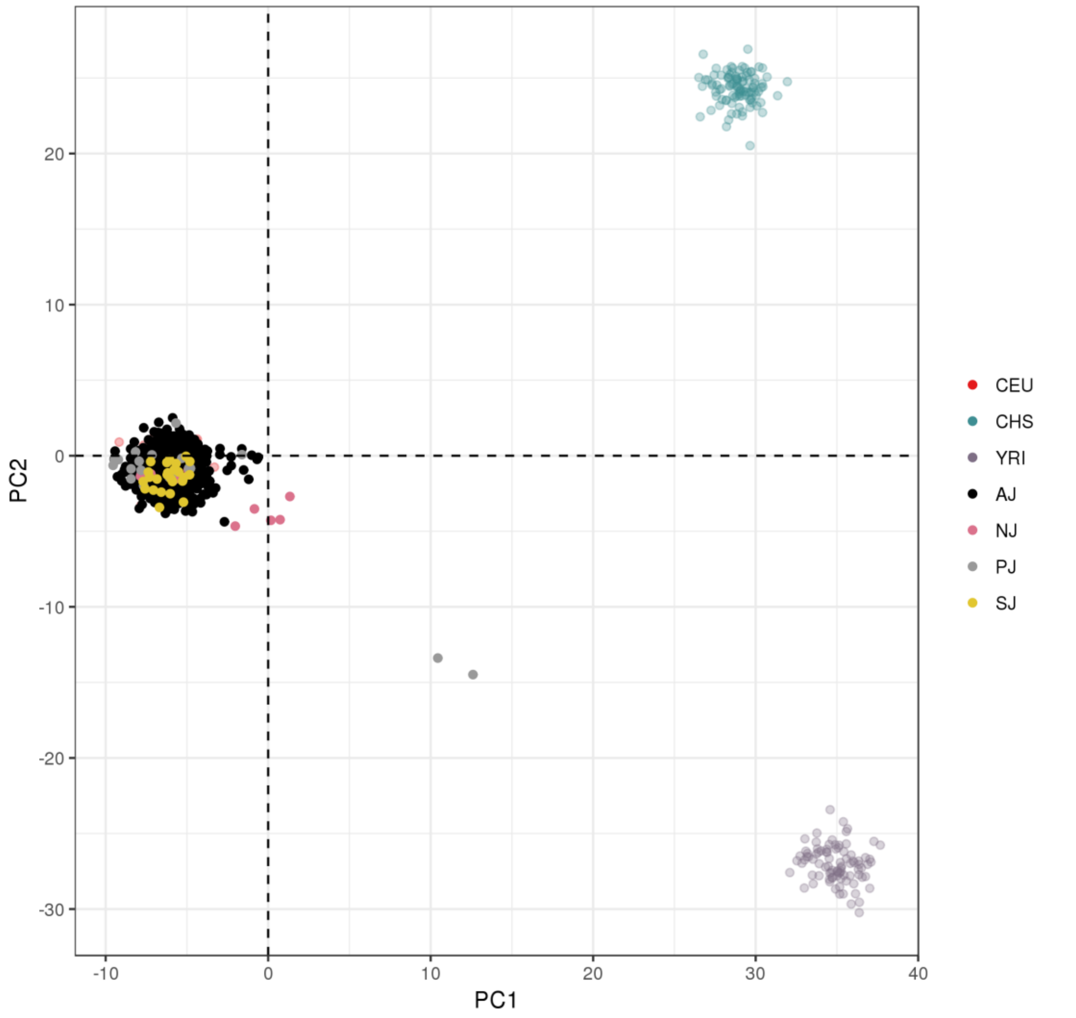

Supplementary Figure 3

B

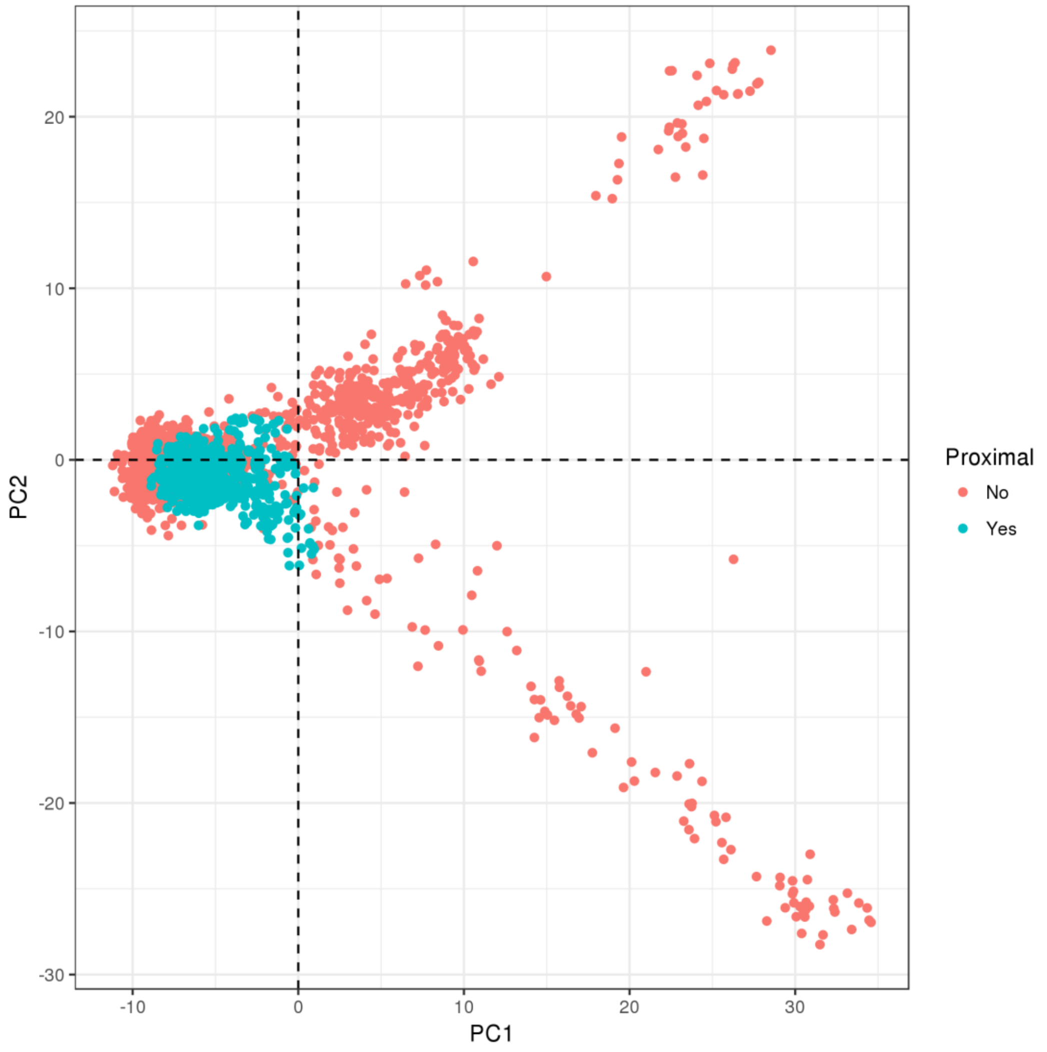

https://doi.org/10.1007/s00439-018-1927-7

<u>Supplementary Figure 4</u>

Quantile-Quantile (QQ) plots for Combined Multivariate Collapsing gene burden tests with a case-control ratio of one. (A) Inflammatory bowel disease. (B) Crohn's disease. For both, there is notable inflation, presumably due to the use of imperfectly matched ancestry controls. In (A), the gene *TAPBP* has a small p-value; however, on decomposition of the logistic regression coefficients, the p-value for the contribution of rare variants is non-significant and additional data would be required to further assess the significance of this finding.

A

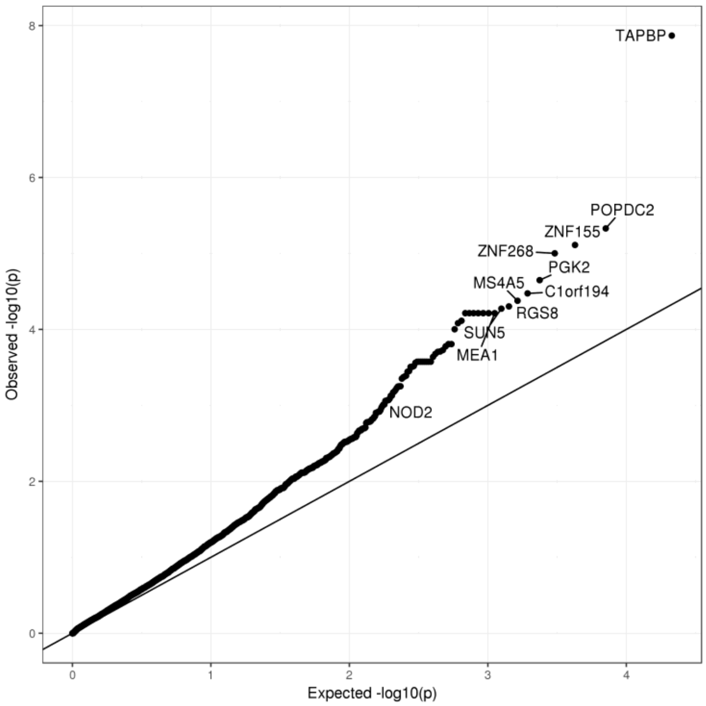

B

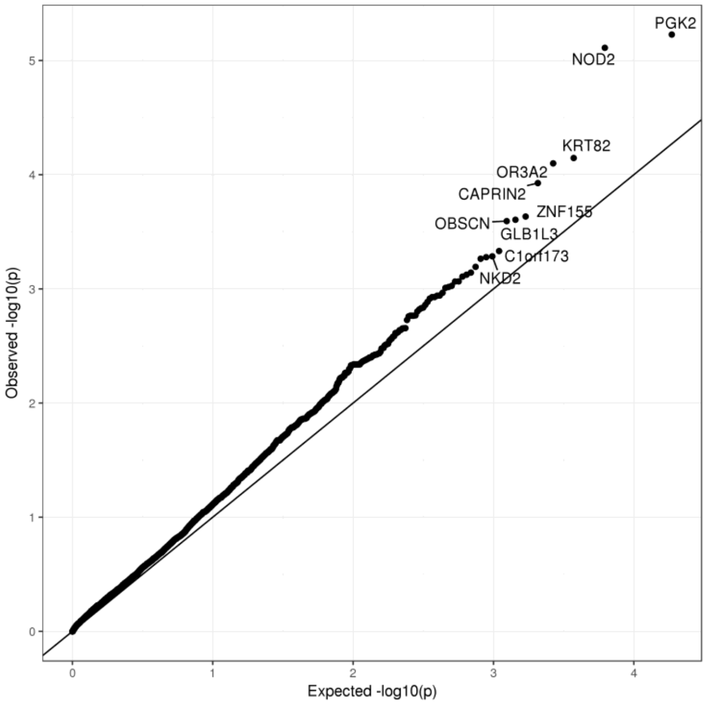

https://doi.org/10.1007/s00439-018-1927-7

Supplementary Figure 5

Results from non-parametric linkage analysis. Log odds (LOD) is plotted against genetic position. The dashed horizontal line is at LOD 1.5. (A) Inflammatory bowel disease, LOD ≥ 1.5 on chromosomes 9 and 13. (B) Crohn's disease, LOD ≥ 1.5 on chromosome 16.

A

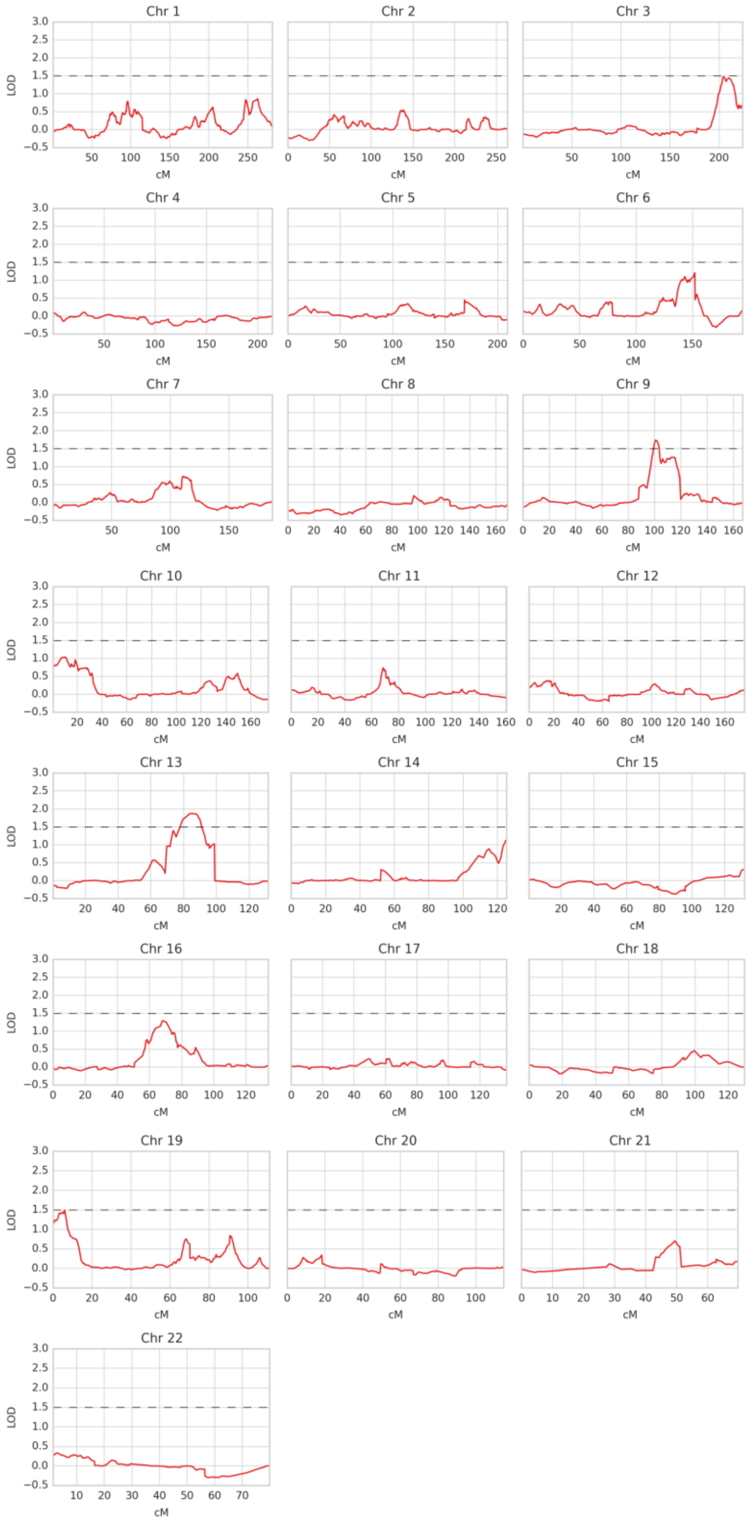

Supplementary Figure 5

B

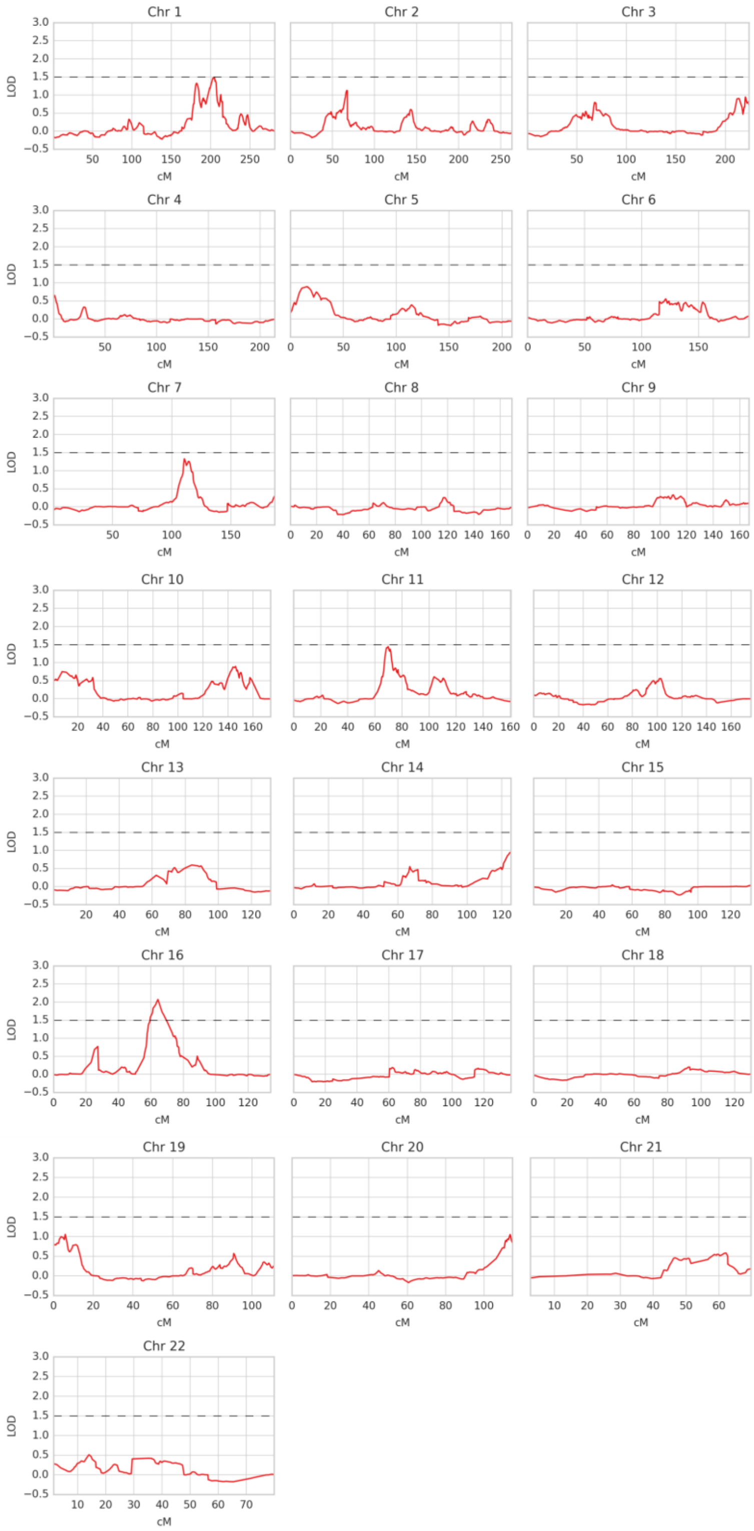

https://doi.org/10.1007/s00439-018-1927-7

Supplementary Figure 6

Pedigrees showing the segregation of the nine variants prioritised based on their occurrence in at least 75% of the affected individuals and in ≤ 1/3 of the unaffected siblings or offspring of affected individuals (Table 2). For each of the four families harbouring the variants, the name of the gene is shown on the left and the genotypes are indicated below each individual in the order of the genes listed where 0 denotes homozygosity for the reference allele and X denotes heterozygosity. No individuals were homozygous for the alternate allele. As per Figure 1, affected individuals are shown by filled symbols and their phenotype (IBD, CD or UC) is indicated. Individuals subjected to exome sequencing are labelled WES.

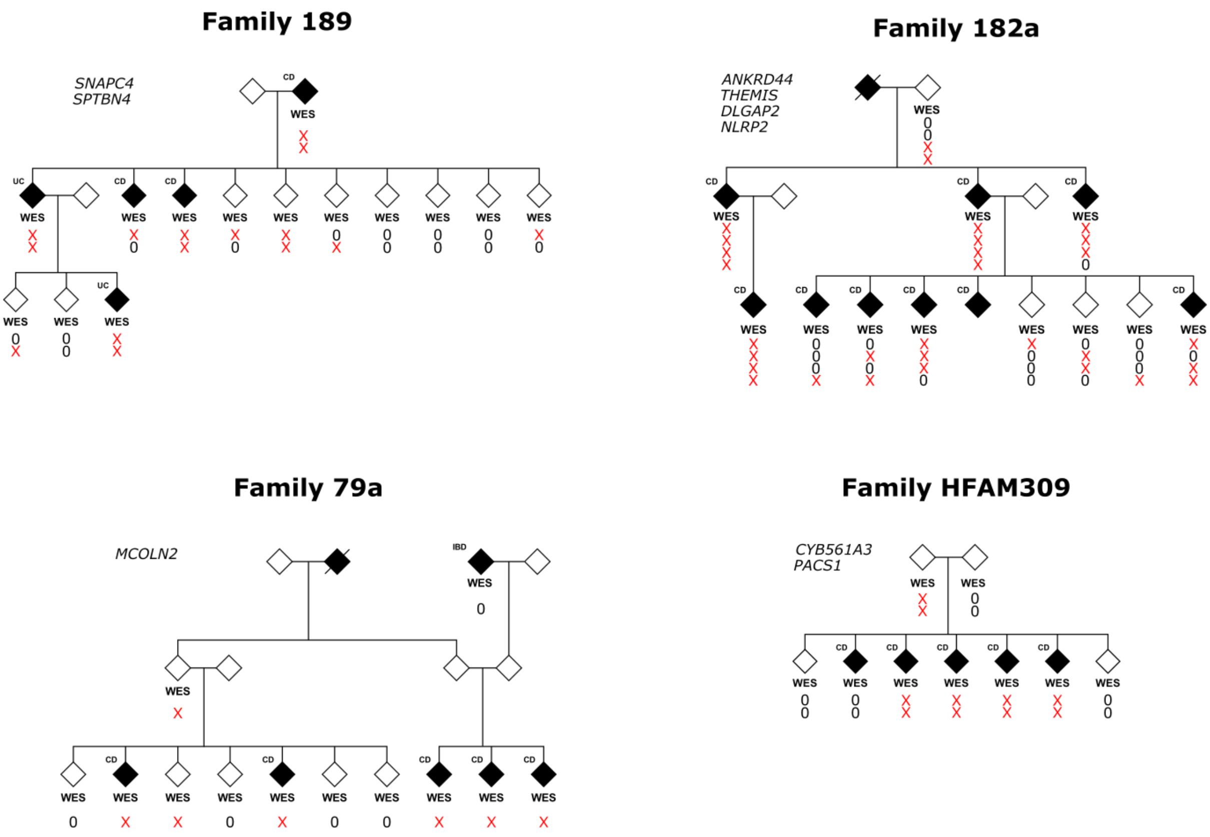

Supplement: Supplementary file 1 — Supplementary Table Legends and Figures (PDF 931 KB) [file 439_2018_1927_MOESM1_ESM.pdf]
